# Activation of aryl hydrocarbon receptor signaling by a novel agonist ameliorates autoimmune encephalomyelitis

**DOI:** 10.1371/journal.pone.0215981

**Published:** 2019-04-26

**Authors:** Alzahrani Abdullah, Mohammed Maged, Ibrahim Hairul-Islam M., Alwassil Osama I., Habash Maha, Alfuwaires Manal, Hanieh Hamza

**Affiliations:** 1 Biological Sciences Department, College of Science, King Faisal University, Hofuf, Saudi Arabia; 2 Department of Pharmaceutical Sciences, College of Clinical Pharmacy, King Faisal University, Hofuf, Saudi Arabia; 3 Department of Pharmacognosy, Faculty of Pharmacy, University of Zagazig, Zagazig, Egypt; 4 Department of Pharmaceutical Sciences, College of Pharmacy, King Saud bin Abdulaziz University for Health Sciences, Riyadh, Saudi Arabia; 5 College of Pharmacy, Aqaba University of Technology, Aqaba, Jordan; 6 Department of Biological Sciences, College of Science, Al-Hussein Bin Talal University, Ma’an, Jordan; 7 Department of Medical Analysis, Aisha Bint Al-Hussein College for Nursing and Health Sciences, Al-Hussein Bin Talal University, Ma’an, Jordan; University of South Carolina School of Medicine, UNITED STATES

## Abstract

**Background:**

Multiple sclerosis (MS) is a widespread neurological autoimmune disease that includes episodes of demyelination in the central nervous system (CNS). The accumulated evidence has suggested that aryl hydrocarbon receptor (Ahr), a ligand-activated transcription factor, is a promising treatment target for MS. Thus, the current study aimed to identify a novel Ahr ligand with anti-inflammatory potential in experimental autoimmune encephalomyelitis (EAE).

**Methods:**

An *in silico* analysis was carried out to predict interactions between Ahr and potential natural ligands. The effects of a predicted interaction were examined *in vitro* using CD4^+^ T cells under T helper17 (Th17) cell-polarizing conditions and lipopolysaccharide (LPS)-stimulated macrophages. Silencing Ahr and microRNA (miR)-132 was achieved by electroporation. Myelin oligodendrocyte glycoprotein (MOG)_35-55_ and the adoptive transfer of encephalitogenic CD4^+^ T cells were used to induce EAE.

**Results:**

Molecular docking analysis and *in vitro* data identified gallic acid (GA) as a novel Ahr ligand with potent activation potential. GA induced the expression of Ahr downstream genes, including cytochrome P450 family 1 subfamily A member 1 (*Cyp1a1*) and the miR-212/132 cluster, and promoted the formation of the Ahr/Ahr nuclear translocator (Arnt) complex. *In vivo*, GA-treated mice were resistant to EAE and exhibited reduced levels of proinflammatory cytokines and increased levels of transforming growth factor-β (TGF-β). Furthermore, GA reduced infiltration of CD4^+^CD45^+^ T cells and monocytes into the CNS. The anti-inflammatory effects of GA were concomitant with miR-132-potentiated cholinergic anti-inflammation and the regulation of the pathogenic potential of astrocytes and microglia. Inducing EAE by adoptive transfer revealed that CD4^+^ T cells were not entirely responsible for the ameliorative effects of GA.

**Conclusion:**

Our findings identify GA as a novel Ahr ligand and provide molecular mechanisms elucidating the ameliorative effects of GA on EAE, suggesting that GA is a potential therapeutic agent to control inflammation in autoimmune diseases such as MS.

## Introduction

Multiple sclerosis (MS) is the most common neurological autoimmune disease of the central nervous system (CNS). MS patients present variable patterns of relapsing remittance characterized by intermittent exacerbations. Such exacerbations and disease progression have often been reduced with disease-modifying therapies [1, 2]. However, some of these therapies exert negative side effects [3].

The immune response in autoimmunity is regulated by both genetic and environmental factors. Although significant progress has been achieved in identifying the genetic control of MS pathogenesis, limited information is available about the contribution of environmental factors [4]. In this context, aryl hydrocarbon receptor (Ahr) represents a valuable model to investigate therapeutic immunomodulation by natural ligands. Ahr, a member of the basic helix-loop-helix (bHLH) family, is implicated in several events of the immune response and autoimmunity [5–7]. It is activated by a variety of exogenous ligands from the diet and environment [8, 9]. Therefore, Ahr signaling integrates the effects of the environment and metabolism on the immune response [10]. In response to ligation, Ahr forms a heterodimer with Ahr nuclear translocator (Arnt) and translocates into the nucleus to induce several downstream genes such as cytochrome P450 family 1 subfamily A member 1 (*Cyp1a1*) [8, 9]. We have also identified the microRNA (miR)-212/132 cluster as a downstream gene of Ahr that mediates some of the immunomodulatory properties of Ahr in autoimmunity [11, 12].

Several studies have introduced endogenous and exogenous ligands that interact with Ahr to attenuate the inflammatory response in animal models of autoimmunity [13, 14]. Two of the potential mechanisms of the reported therapeutic potential of Ahr ligands is promoting the generation of regulatory T (Treg) cells and suppressing proinflammatory mediators. For example, 2-(1' H-indole-3'-carbonyl)-thiazole-4-carboxylic acid methyl ester (ITE), an endogenous Ahr ligand, ameliorates autoimmune inflammation by inducing Treg cells and reducing proinflammatory cytokine levels and the macrophage frequency in experimental models of colitis [13] and uveoretinitis [15]. 3,3'-Diindolylmethane (DIM), a dietary Ahr ligand, shifts the balance among T helper 2 (Th2)/Th17/Treg cells toward Treg cells to ameliorate colitis [16]. Moreover, norisoboldine, a natural Ahr ligand identified by means of *in silico*, *in vitro* and *in vivo* investigations, alleviates autoimmune inflammation by inducing the generation of Treg cells and suppressing proinflammatory cytokines in experimental models of arthritis [17] and colitis [18].

In an experimental model of MS, DIM- and indole-3-carbinol (I3C)-activated Ahr were shown to inhibit clinical symptoms and cellular infiltration within the CNS by promoting the generation of Treg cells while suppressing myelin oligodendrocyte glycoprotein (MOG)-specific Th17 cells [19]. Laquinimod, an oral drug being evaluated for the treatment of MS, attenuates experimental autoimmune encephalomyelitis (EAE) by inducing the generation of Treg cells and suppression of proinflammatory cytokines in an Ahr-dependent fashion [20]. Furthermore, 2,3,7,8-tetrachlorodibenzo-p-dioxin (TCDD) activates Ahr to induce miR-132-mediated cholinergic anti-inflammatory processes in EAE [11]. It has been recently shown that type I interferons (IFN-Is) in combination with indole, indoxyl-3-sulfate (I3S), indole-3-propionic acid (IPA) and indole-3-aldehyde (IAld) activate Ahr signaling in astrocytes to suppress CNS inflammation in EAE [21].

In the current study, we used a combination of *in silico*, *in vitro* and *in vivo* approaches to identify a natural Ahr ligand with therapeutic potential in EAE. For the first time, we introduce gallic acid (GA) as a novel Ahr ligand of natural origin and provide a mechanistic explanation for the anti-inflammatory properties of GA.

## Materials and methods

### *In silico* analysis

The alignment of the Ahr PAS-A sequence (UniProt; P30561) with several orthologues was performed by ClustalX 2.0 [22]. The Ahr PAS-A three-dimensional (3D) structure was obtained from the RCSB Protein Data Bank (ID: 4M4X; http://www.rcsb.org), and the chemical structure of GA was obtained from the PubChem database (CID_370; www.ncbi.nlm.nih.gov/pccompound). The sequence of mouse PAS-B (NP_038492.1) was obtained from NCBI (https://www.ncbi.nlm.nih.gov/protein/). Modeling of the 3D structure was established for the stereochemical value by SAVES version v5.0 software (https://services.mbi.ucla.edu/SAVES/), and the 3D structure of PAS-B was coordinated by using PS2 software (http://www.ps2.life.nctu.edu.tw/) [23]. The confirmation of model overlap with the retrieved human C-terminal PAS domain of HIF2a was performed by using SALIGN software (https://modbase.compbio.ucsf.edu/salign/).

The molecular docking simulation of GA against Ahr domains was performed by using SYBYLX 2.1 (Tripos Associates Inc.). The docking conditions included applying the ChemPLP scoring function within the genetic algorithm docking program GOLD 5.2 (Cambridge Crystallographic Data Centre). Based on the obtained scores and the molecular orientation within the binding pocket, the molecule with the best score was selected and merged into the receptor. The model-ligand complexes were energetically adjusted using the Tripos Force Field (Gasteiger-Hückel charges, distance-dependent dielectric constant = 4.0) to optimize the interactions between the ligand and receptor within the binding cavity.

### Mice and ethics statement

The female 6-8-week-old C57BL/6 mice for all experiments were purchased originally from Charles River Laboratories and maintained under specific pathogen-free conditions. All *in vivo* and *in vitro* experiments were performed in accordance with protocols approved by the Research Ethics Committee (KFU-REC/2018-3-1) of King Faisal University, Saudi Arabia. The humane endpoints included ≥ 25% body weight loss, paresis or forelimbs paralysis for 24 h.

### Cell isolation and differentiation

Naïve T cells were isolated from the spleen by using a MACS CD4^+^CD62L^+^ isolation kit (Miltenyi Biotec). The differentiation of Th17, Treg and type 1 regulatory T (Tr1) cells was induced as previously described [6, 24]. The purified CD4^+^CD62L^+^ T cells were cultured in the presence of Dynabeads Mouse T-Activator CD3/CD28 (Invitrogen). The cell culture contained IL-6 (30 ng/mL), TGF-β1 (4 ng/mL), anti-IFN-γ and anti-IL-4 (10 μg/mL) to generate Th17 cells; TGF‐β1 (4 ng/mL) and IL‐2 (20 U/mL) to generate Treg cells; and TGF‐β1 (2 ng/mL) and IL‐27 (30 ng/mL) to generate Tr1 cells. All recombinant cytokines were purchased from R&D Systems, and anti-IFN-γ and anti-IL-4 antibodies were obtained from BioLegend. Peritoneal macrophages were stimulated with 0.5 μg/mL lipopolysaccharide (LPS; Sigma-Aldrich). GA, DIM (30 μmol/L) and phytohemagglutinin (PHA; 5 μg/mL) were purchased from Sigma-Aldrich. To isolate microglia, astrocytes and monocytes from the CNS, tissue samples were prepared using the MACS Neuronal Dissociation Tissue Kit, and then cells were isolated using MACS isolation kits. The absolute number of Ly-6C^hi^ monocytes was assessed by flow cytometry. To isolate CD4^+^ and CD4^+^CD45^+^ cells from the CNS, mononuclear cells were separated from the CNS using a gradient comprising 30% and 70% Percoll solutions (GE Healthcare) as previously described [25], and then the cells were isolated using MACS CD4^+^ and CD45^+^ cell isolation kits following the manufacturer's instructions.

### Quantitative real-time PCR

cDNA was synthesized by using a TaqMan reverse transcription kit and amplified by using a ViiA7 system and TaqMan gene expression assays for *Cyp1a1* (Mm00487218_m1), *Rorc* (Mm01261022_m1), *Foxp3* (Mm00475162_m1), *Il6* (Mm00446190_m1), *Il10* (Mm01288386_m1), *Ccl2* (Mm00441242_m1), *Csf2* (Mm01290062_m1), *Nos2* (Mm00440502_m1) and *Gapdh* (Mm99999915_g1) for coding genes or TaqMan microRNA assays for has-miR-132 (ID: 000457), has-miR-212 (ID: 002551) and *RNU6B* (ID: 001093) for noncoding genes. Kits, probes and reagents were obtained from Applied Biosystems. The relative expression of mRNAs was calculated by the ΔΔCt method.

### Transfection and luciferase activity

CD4^+^CD62L^+^ cells and peritoneal macrophages were transfected with oligonucleotides using Primary Cell Nucleofector kits and a 4D-Nucleofector system (Lonza). An siRNA specific for Ahr (siAhr, 75 nmol/L), a nonspecific siRNA (siNS, 75 nmol/L), an antisense (as)-miR-132 (250 μmol/L) and a scrambled control (250 μmol/L) were obtained from Ambion. qPCR and immunoblotting were used to confirm transfection efficiencies. Reporter plasmid and luciferase activities in the presence of siAhr were assessed following a modified method described elsewhere [26]. A 4,553 bp fragment of the mouse miR-212/132 promoter [27] was amplified using the following oligonucleotide primers: forward, 5'-AGATCGCCGTGTAATTCTAGAGGGAAGGTTCTGTCTTCAAATGAGGAACTC-3' and reverse, 5'-TTCTCGCCACCTTAGGCAGCGATACCCGGCCGCCCCGACTCTAGA-3'. The purified PCR product was cloned into the XbaI restriction site of the pGL3 plasmid (Promega) using the In-Fusion HD Cloning Kit (Clontech). The empty or miR-212/132 promoter-encoding pGL3 vector (100 ng) was cotransfected with siNS or siAhr into CD4^+^CD62L^+^ cultured under Th17 cell-conditions and macrophages by electroporation. Luciferase activity was quantified using the Dual-Luciferase Reporter System (Promega) following the manufacturer's instructions.

### Protein quantification

Cell lysates were prepared using the RIPA Lysis Buffer System and fractionated by SDS-PAGE. Target proteins in the lysates and in immunoprecipitation eluates were detected by rabbit polyclonal antibodies specific for Cyp1a1, Ahr, Arnt and AChE (dilution; 1:500), mouse monoclonal antibodies specific for β-actin (dilution; 1:1,000) and the corresponding horseradish peroxidase (HRP)-conjugated secondary antibodies (1:5,000). The Lysis Buffer System and antibodies were purchased from Santa Cruz Biotechnology. Band intensity was quantified by ImageJ software (version 1.48; https://imagej.nih.gov/ij/download.html). To quantify the serum and supernatant cytokine levels, ELISA kits for IL-17a, IL-6, TNF-α, TGF-β (Invitrogen), and IL-10 (GenWay) were used following the manufacturer’s instructions.

### Flow cytometry

Isolated CD4^+^ T cells were stimulated with 50 ng/mL phorbol 12-myristate 13-acetate (Sigma-Aldrich) and 800 ng/mL ionomycin (Sigma-Aldrich) for 5 h, with Protein Transport Inhibitor (Invitrogen) added for the final 2 h. An Intracellular Staining kit (Life Technologies) and phycoerythrin (PE)-conjugated anti-IL-17 antibodies (eBioscience) were used following the manufacturer’s instructions. Foxp3 was stained by using a Foxp3 Staining kit (Invitrogen) including fluorescein isothiocyanate (FITC)-conjugated anti-Foxp3 according to the manufacturer’s instructions. For surface staining, PerCP-Cy5.5-conjugated anti-CD4 antibodies and FITC-conjugated anti-CD45 antibodies from eBioscience were used. The analysis was performed by using a FlowSight system (Amnis).

### EAE models

Mice were immunized with MOG_35-55_ (150 μg; Peptide International) emulsified in complete Freund's adjuvant (CFA; Sigma-Aldrich) containing 10 mg/mL heat‐killed *Mycobacterium tuberculosis* H37Ra (Difco Laboratories). The mice were injected intraperitoneally with pertussis toxin (List Biological Laboratories; 500 ng) on days 0 and 2. The adoptive transfer of encephalitogenic CD4^+^ T cells isolated 9 days after immunization was also used to induce EAE. The encephalitogenic CD4^+^ T cells were restimulated with MOG_35‐55_ (30 μg/mL) and IL‐23 (20 ng/mL; R&D Systems) for 72 h before being injected in naïve mice at a dose of 1×10^7^ cell/mouse. Scoring of clinical symptoms was as follows: 0, no clinical signs; 1, hind limb weakness or limp tail; 2, paralyzed hind limb; 3, paralyzed forelimb; 4, complete paralysis; and 5, death. The mice were injected intraperitoneally (i.p.) with GA (2 mg/day) or vehicle (corn oil) for 10 days starting one day before the MOG_35-55_ immunization.

### Statistical analysis

Data were pooled and are shown as the mean ± SD from three independent experiments performed in triplicate using three mice per experiment unless otherwise indicated. The mean values were tested for statistical significance by one‐way ANOVA. The statistical significance of the differences between EAE clinical scores was analyzed by two‐way ANOVA, and the *X*^2^ test was used to test differences between EAE incidence rates (%). *p < 0.05.

## Results

### GA is a potential Ahr ligand

**Fig 1:**
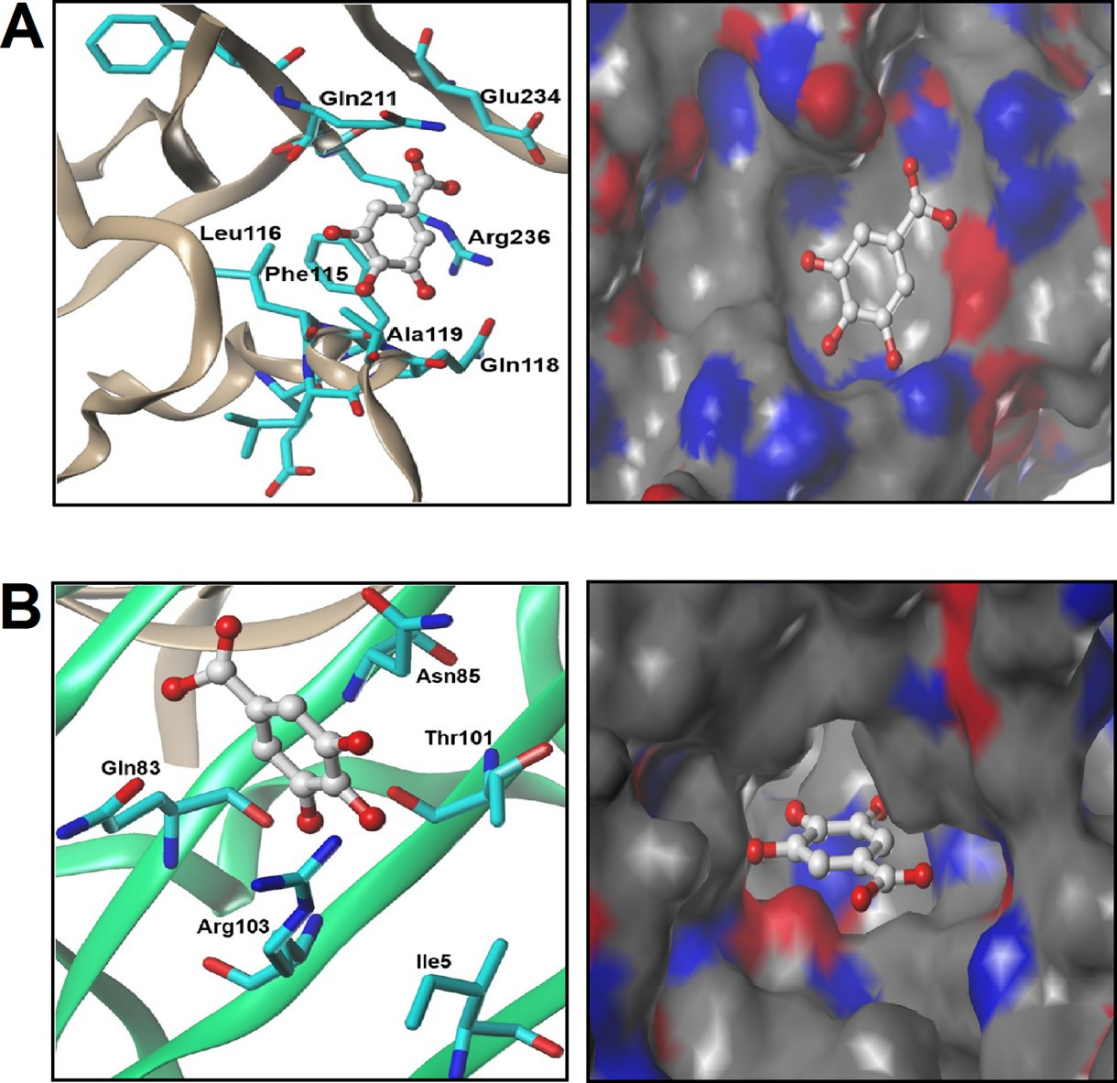
GA has binding potential to Ahr domains. The binding mode of docked GA (ball and stick model) in mouse Ahr (A) PAS-A and (B) PAS-B binding sites. The labelled residues in left panel represent the major interacting amino acids in the binding site that interact with GA. Right panel represents contour maps for the binding site of GA showing polar areas (blue and red) and hydrophobic areas (gray).

In an attempt to identify a novel Ahr ligand, we first ran an *in silico* screening experiment using molecular docking simulation. Ahr, like other members of the bHLH family, contains PAS-A and PAS-B domains that are involved in transforming Ahr into its transcriptionally active form by the formation of the Ahr/Arnt complex. Therefore, both domains were used to perform docking simulation with a customized screening library that included natural aromatic hydrocarbons. Among the tested molecules, GA formed a relatively strong interaction with Ahr PAS-A residues with a binding score of 39.4 ChemPLP. It formed five hydrogen bonds with the PAS-A residues Phe115, Leu116, Ala119, Gln211 and Glu234 (Fig 1A and 1B). Furthermore, GA formed an ionic interaction between its negatively charged carboxylate group and the positively charged guanidine moiety of Arg236 (Fig 1A).

The PAS-B domain of Ahr plays a critical role in Ahr activation by serving as a ligand-binding domain. Docking analysis revealed that GA formed a relatively strong interaction with PAS-B residues with a binding score of 46.24 ChemPLP. GA formed one hydrogen bond with Gln83, one hydrogen bond with Asn85 and two hydrogen bonds with Arg103 (Fig 1B). In addition, a hydrophobic interaction was predicted to occur between Thr101 and Ile5. Taken together, these observations suggest that GA is a potential Ahr ligand. The 3D coordinates of PAS-B, a Ramachandran plot and the analysis of G-factor parameters are presented in S1 Fig.

### GA induces the expression of Ahr downstream genes

Ahr classically forms a heterodimeric complex with Arnt and translocates to the nucleus to induce the transcription of downstream genes such as *Cyp1a1*. Therefore, we first examined the effect of GA on *Cyp1a1* gene expression in naïve CD4^+^ T cells cultured under Th17 conditions cells and LPS-stimulated macrophages, the cells implicated in EAE pathogenesis. The efficiency of the effector CD4^+^ cells-inducing milieus and the stimulation of macrophages were confirmed (S2 Fig).

**Fig 2:**
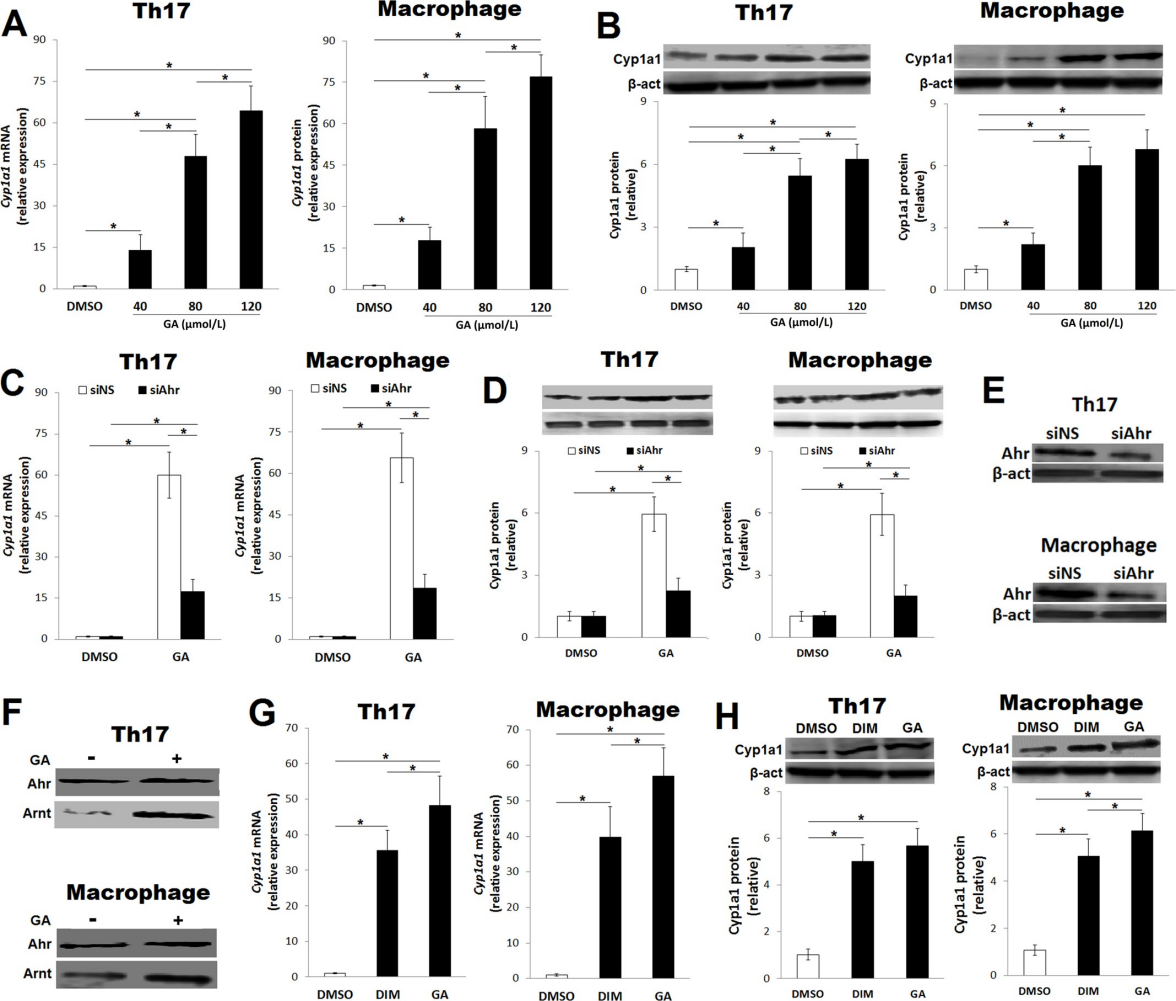
GA induces gene expression of *Cyp1a1* and formation of Ahr/Arnt complex. The CD4^+^CD62L^+^ T cells were isolated from the spleen and cultured under Th17-polarizing conditions, and peritoneal macrophages were cultured in presence of LPS. The expression of *Cyp1a1* mRNA was assessed by quantitative real‐time PCR and normalized to *Gapdh* mRNA, and protein level was assessed by immunoblot using Actin as a loading control. (A) Relative expression of *Cyp1a1* mRNA at 12 h in CD4^+^CD62L^+^ T cells cultured under Th17-polarizing conditions and macrophages in presence of GA (40–120 μmol/L) compared to vehicle (DMSO). (B) Immunoblot and relative protein level of Cyp1a1 at 48 h in CD4^+^CD62L^+^ T cells cultured under Th17-polarizing conditions and macrophages in presence of GA (40–120 μmol/L) compared to DMSO. (C) Relative expression of *Cyp1a1* mRNA at 12 h in CD4^+^CD62L^+^ T cells cultured under Th17-polarizing conditions and macrophages electroporated with Ahr siRNA (siAhr) or non-specific siRNA (siNS) in presence of GA (80 μmol/L) compared to DMSO. (D) Immunoblot and relative protein level of Cyp1a1 at 48 h in CD4^+^CD62L^+^ T cells cultured under Th17-polarizing conditions and macrophages electroporated with siAhr or siNS in presence of GA (80 μmol/L) compared to DMSO. (E) Efficiency of siAhr in CD4^+^CD62L^+^ T cells cultured under Th17-polarizing conditions and macrophages was confirmed by immunoblot. (F) Detection of Arnt protein by immunoblot in the eluates pulled down by Ahr antibodies using CD4^+^CD62L^+^ T cells cultured under Th17-polarizing conditions and macrophages. (G and H) Relative expression of (G) *Cyp1a1* mRNA and (H) protein in presence of DIM (25 μmol/L) or GA (80 μmol/L) in CD4^+^CD62L^+^ T cells cultured under Th17-polarizing conditions and macrophages compared to DMSO. Data were pooled from three independent experiments with three mice per experiment and shown as mean ± SD. *p < 0.05, (one‐way ANOVA); horizontal bars denote statistical comparison.

As depicted in Fig 2A and 2B, GA (40–120 μmol/L) upregulated *Cyp1a1* mRNA and protein expression in differentiating Th17 cells and macrophages in a concentration-dependent fashion. Studying the effects of GA on cell viability showed a reduction in cell viability at a dose of 120 μmol/L GA (S3 Fig). Thus, 80 μmol/L GA was used hereafter. The depletion of Ahr by RNA interference abolished the enhancing effects of GA (80 μmol/L) on *Cyp1a1* gene expression (Fig 2C and 2D), suggesting an Ahr-dependent mode of action. The efficiency of Ahr depletion by a specific siRNA (siAhr) was confirmed (Fig 2E).

Because the dimerization of Ahr with Arnt is required to activate the transcriptional activity of Ahr, we examined the formation of the heterodimer by immunoprecipitation using anti-Ahr antibodies. Detection of the Arnt protein in an eluate confirmed the formation of the Ahr/Arnt complex in GA-treated differentiating Th17 cells and macrophages (Fig 2F). Finally, we compared the effects of GA and DIM, a well-known natural ligand of Ahr, on the expression of the *Cyp1a1* gene. Interestingly, the *Cyp1a1* mRNA and protein levels were significantly higher with GA treatment than DIM treatment (Fig 2G and 2H).

**Fig 3:**
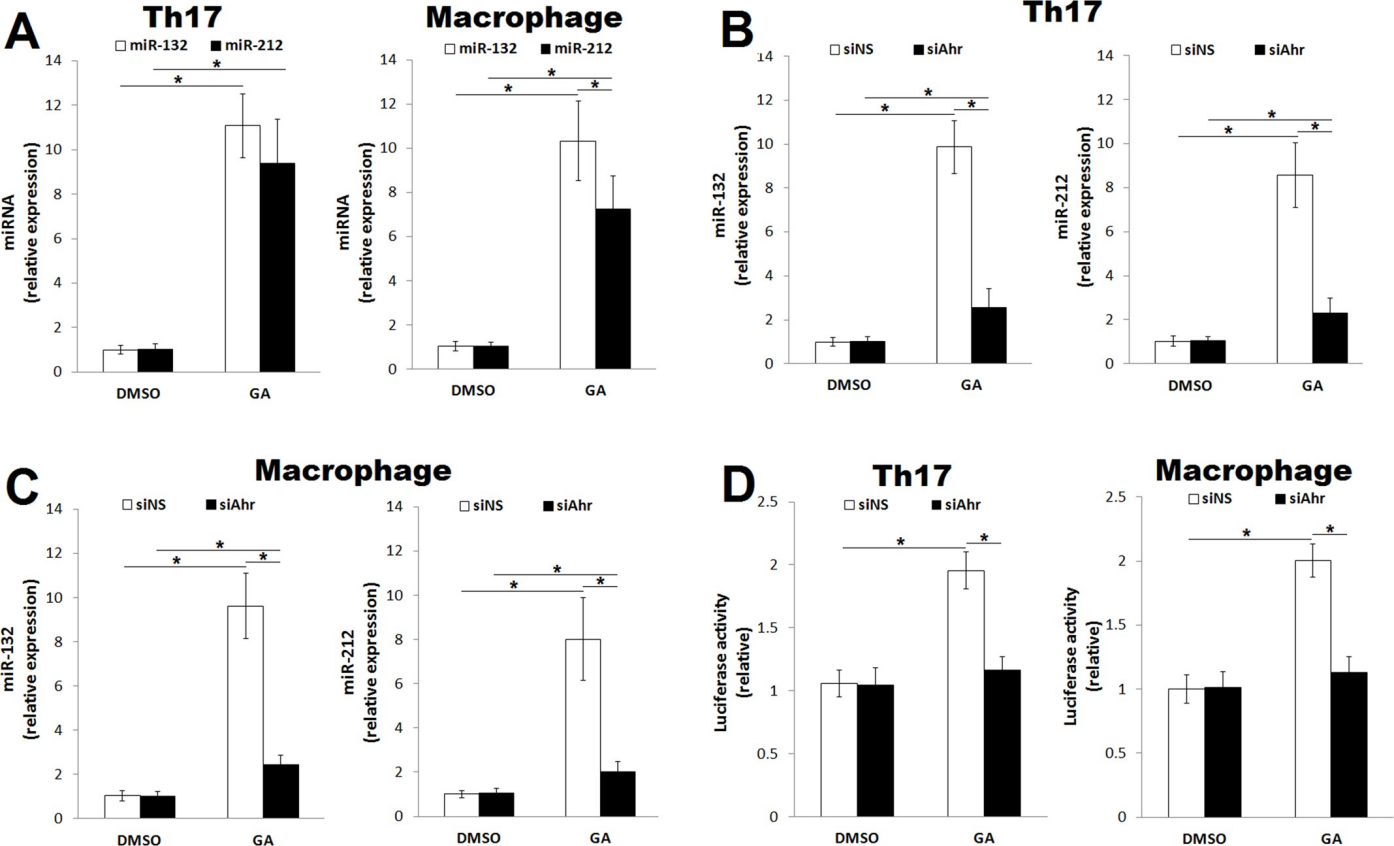
GA-activated Ahr induces the expression of miR-212/132 cluster. The CD4^+^CD62L^+^ T cells were isolated from the spleen and cultured under Th17-polarizing conditions, and peritoneal macrophages were stimulated with LPS. The expression of miR-132 and miR-212 were assessed by quantitative real‐time PCR and normalized to *RNU6B* mRNA. (A) Relative expression of miR-132 and miR-212 in CD4^+^CD62L^+^ T cells cultured under Th17-polarizing conditions (72 h) and macrophages (36 h) in presence of GA (80 μmol/L) compared to vehicle (DMSO). (B) Relative expression of miR-132 and miR-212 in CD4^+^CD62L^+^ T cells cultured under Th17-polarizing conditions (72 h) and electroporated with Ahr siRNA (siAhr) or non-specific siRNA (siNS) compared to DMSO. (C) Relative expression of miR-132 and miR-212 in macrophages (36 h) electroporated with siAhr or siNS compared to DMSO. (D) Relative luciferase activity of miR-212/132 promoter reporter cotransfected with siNS or siAhr into CD4^+^CD62L^+^ T cells cultured under Th17-polarizing conditions and macrophages compared to DMSO. Data were pooled from three independent experiments with three mice per experiment and shown as mean ± SD. *p<0.05, (one‐way ANOVA); horizontal bars denote statistical comparison.

We previously identified the miR-212/132 cluster as a downstream gene of ligand-activated Ahr [11, 12]. Therefore, we tested whether GA-activated Ahr induces the expression of miR-132 and miR-212. As predicted, GA treatment (80 μmol/L) induced the expression of miR-132 and miR-212 in both polarized Th17 cells and macrophages (Fig 3A), and knocking down Ahr expression abrogated these effects (Fig 3B and 3C). To examine whether Ahr has a direct transcriptional activity on miR-212/132, differentiating Th17 cells and macrophages were cotransfected with a reporter plasmid encoding the miR-212/132 promoter and siAhr. As depicted in Fig 3D, GA-activated Ahr induced luciferase activity driven by the miR-212/132 promoter, whereas knocking down Ahr expression abolished this effect, indicating a direct transcriptional activation role for Ahr on miR-212/132. Taken together, these findings suggest that GA is a novel Ahr agonist.

### GA ameliorates EAE

**Fig 4:**
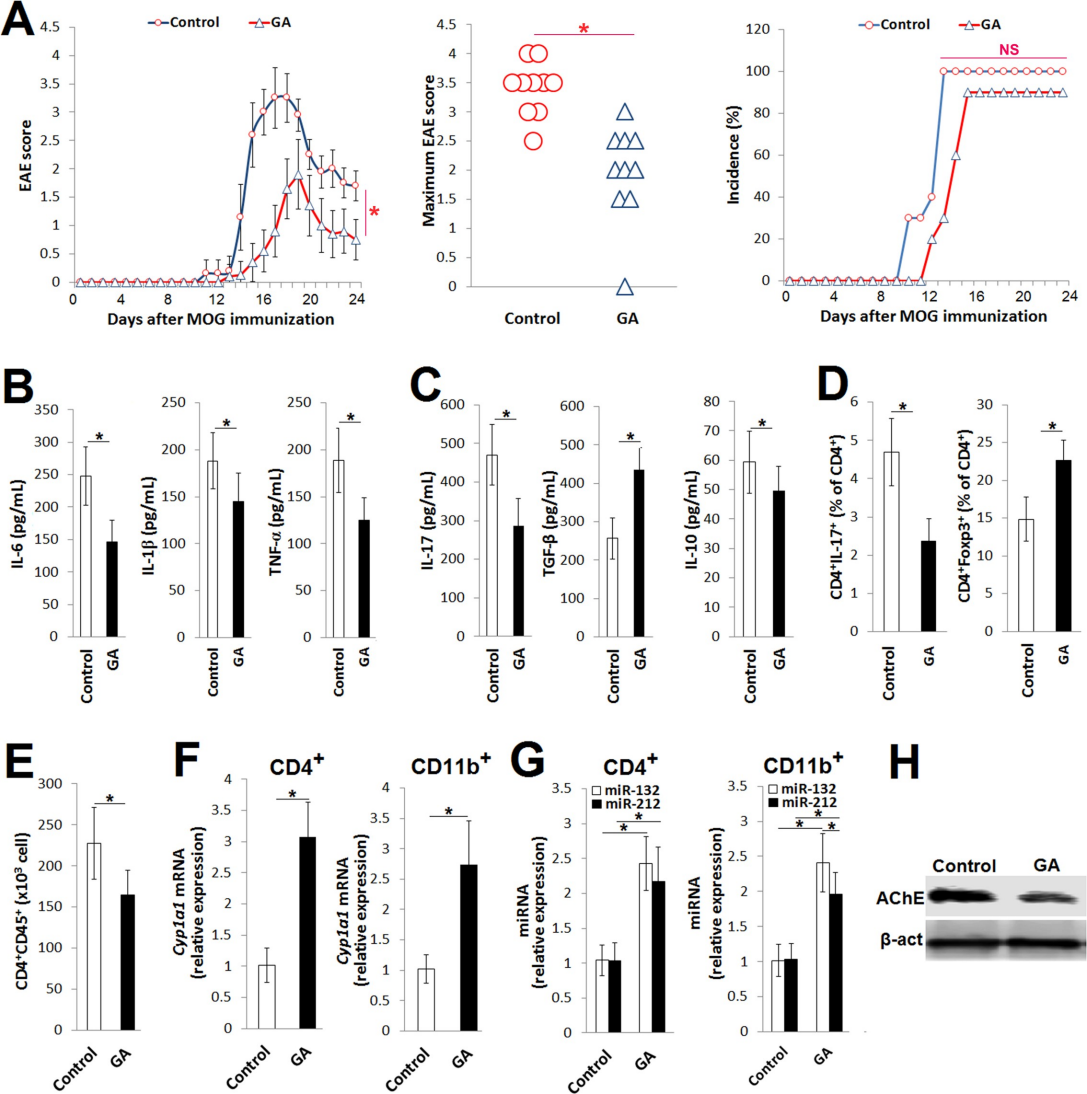
GA-activated Ahr alleviates EAE severity. The mice were immunized with MOG_35-55_ emulsified in CFA. The mRNA expression of *Cyp1a1* and miR-212/132 cluster were assessed in isolated CD4^+^ T cells and CD11b^+^ cells by quantitative real-time PCR and normalized to the mRNAs of *Gapdh* for *Cyp1a1* and *RNU6B* for miRNAs. The levels of cytokines were quantified by ELISA. (A) EAE clinical score, maximum score and incidence (%) of Control EAE (Control) and GA EAE mice, n = 10 each. (B) Serum levels of IL-6, IL-1β and TNF-α in EAE mice 24 days after MOG_35-55_ immunization. (C) Levels of IL-17, TGF-β and IL-10 in culture supernatant of encephalitogenic CD4^+^ T cells restimulated with MOG_35-55_ and IL-23 for 72 h. (D) Frequency (%) of CD4^+^IL-17^+^ and CD4^+^Foxp3^+^ T cells in total CD4^+^ T cells isolated from inguinal lymph nodes 24 days after MOG_35-55_ immunization. (E) Absolute number of CD4^+^CD45^+^ T cells in the CNS 18 days after MOG_35-55_ immunization. (F) Relative expression of *Cyp1a1* mRNA in CD4^+^ T cells and CD11b^+^ cells isolated from spleen 10 days after MOG_35-55_ immunization compared to Control. (G) Relative expression of miR-132 and miR-212 in CD4^+^ T cells and CD11b^+^ cells isolated from spleen 10 days after MOG_35-55_ immunization compared to Control. (H) Representative immunoblot of AChE in spleen WBCs 10 days after MOG_35-55_ immunization. Data were pooled from three independent experiments and shown as mean ± SD. *p<0.05; (A) EAE score, two‐way ANOVA; (A) incidence (%), *X*^2^; (A) maximum score and (B-G), one-way-ANOVA; horizontal bars denote statistical comparison.

To ascertain the biological significance of Ahr activation by GA *in vivo*, we first compared disease severity between control EAE (Control) and GA-treated EAE (GA) mice. Studying the effects of different GA doses on the weight of the spleen and liver showed a significant increase in liver weight at a GA dosage of 4 mg/day (S3 Fig). GA treatment for 10 days, starting one day prior to MOG_35-55_ immunization, attenuated clinical and maximum EAE scores but not disease incidence (Fig 4A). The attenuated EAE symptoms were associated with lower cytokine levels of IL‐6, IL-1β and TNF‐α on day 24 after MOG_35-55_ immunization (Fig 4B). Moreover, the CD4^+^ T cells isolated from the inguinal lymph nodes of the GA mice 9 days after immunization produced less IL‐17 in response to restimulation with MOG_35-55_ than the CD4^+^ T cells from the Control mice (Fig 4C). Notably, GA enhanced the production of TGF-β but decreased that of IL-10 (Fig 4C). Consistent with the ameliorated EAE symptoms and altered levels of IL-17 and TGF-β, a flow cytometry analysis revealed that GA reduced the frequency of CD4^+^IL-17^+^ T cells and increased that of CD4^+^Foxp3^+^ T cells (Fig 4D). Because the infiltration of activated T cells is important for clinical EAE symptoms, we compared the number of CD4^+^CD45^+^ T cells in the CNS between the Control and GA EAE mice on day 12 after immunization. As predicted, the total number of CD4^+^CD45^+^ T cells was significantly lower in the CNS of the GA EAE mice (Fig 4E).

We next examined whether GA induces the transcription of the *Cyp1a1* gene in CD4^+^ T cells and CD11b^+^ macrophages collected from the spleen of EAE mice. In line with the *in vitro* results, the mRNA expression of *Cyp1a1* was upregulated in the examined cells (Fig 4F). Furthermore, GA induced the expression of miR-132 and miR-212 in CD4^+^ T cells and CD11b^+^ macrophages from the spleen of EAE mice (Fig 4G). The activation of Ahr *in vivo* induces acetylcholinesterase (AChE)-targeting miR-132 to potentiate cholinergic anti-inflammatory processes [11, 28]. Interestedly, the AChE protein level was reduced in the spleens from the GA EAE mice (Fig 4H), suggesting that GA could potentiate cholinergic anti-inflammatory processes. This finding was supported by the *in vitro* data showing that GA suppressed the AChE activity in PHA-stimulated CD4^+^ T cells and LPS-stimulated macrophages (S4 Fig). Collectively, these results indicate that the ameliorative effects of GA are at least partially attributed to Ahr activation.

### The ameliorative effects of GA on EAE are not entirely mediated by CD4^+^ T cells

**Fig 5:**
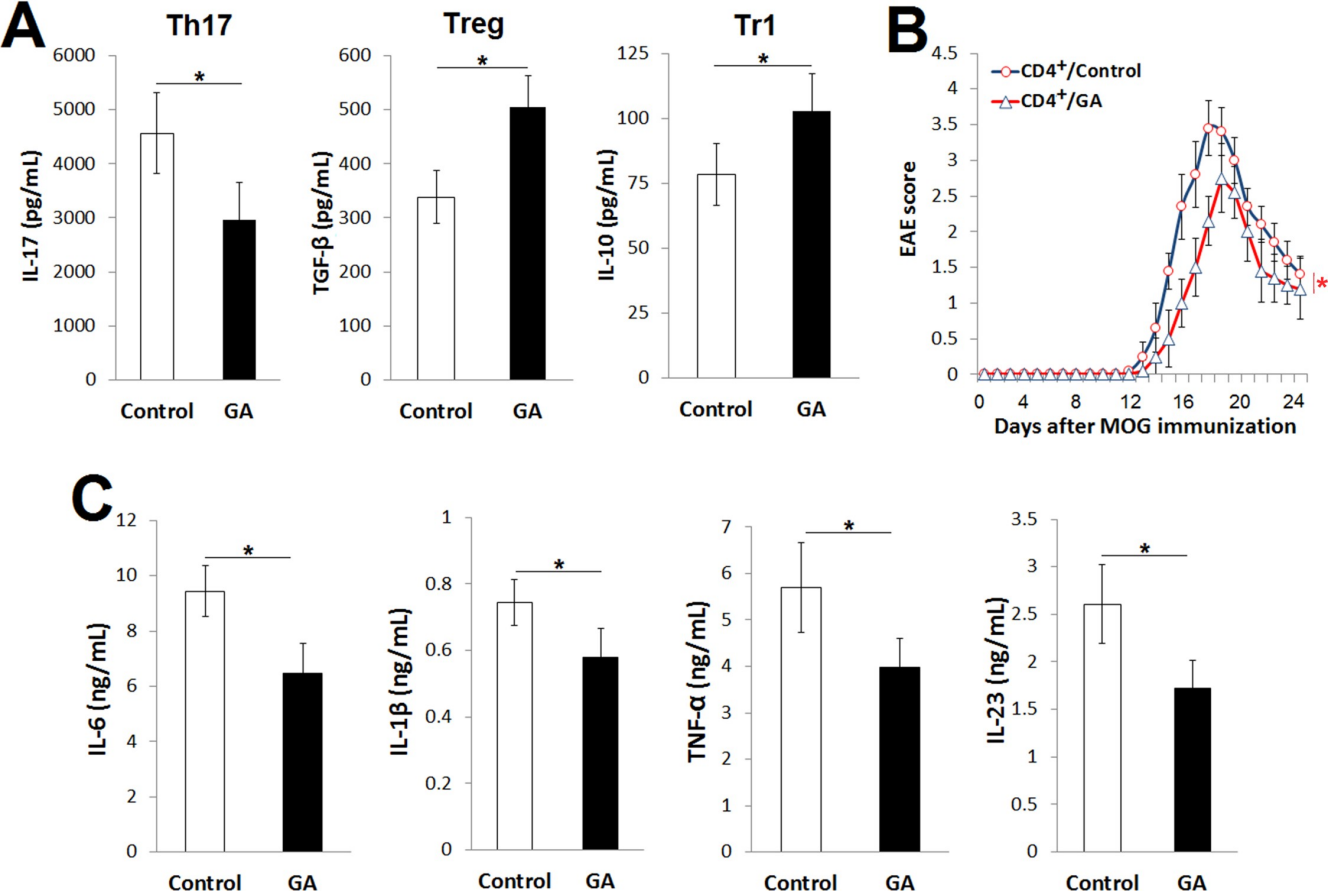
GA alleviates EAE by CD4^+^-dependent and -independent mechanisms. The CD4^+^CD62L^+^ T cells were isolated from the spleen and cultured under Th17-, Treg- or Tr1-polarizing conditions, and peritoneal macrophages were cultured in presence of LPS. (A) Levels of IL-17, TGF-β and IL-10 at 72 h in culture supernatant of polarized Th17, Treg or Tr1cells, respectively. (B) Clinical scores of EAE induced by adoptive transfer of encephalitogenic CD4^+^ T cells from control EAE (Control) or GA EAE mice, *n* = 10 each. (C) Levels of IL-6, IL-1β, TNF-α and IL-23 at 16 h in culture supernatant of macrophages. Data were pooled from three independent experiments with three mice per experiment and shown as mean ± SD. *p<0.05; (A and C), one-way-ANOVA; (B), two-way ANOVA.

Th17 cells, which correlate reciprocally with Treg cells, play a pivotal role in the pathogenesis of EAE. Therefore, we studied the effects of GA on the effector functions of polarized Th17, Treg and Tr1 cells. Consistent with the *in vivo* observations, GA (80 μmol/L) suppressed the production of IL-17 and enhanced that of TGF-β (Fig 5A). However, GA treatment showed a discrepancy in IL-10 production between the *in vitro* and *in vivo* experiments. Subsequently, we studied the intrinsic role of CD4^+^ T cells in EAE. Thus, we induced EAE by adoptively transferring encephalitogenic CD4^+^ T cells isolated from the Control or GA EAE mice. These CD4^+^ T cells were restimulated with MOG_35‐55_ and IL‐23 for 72 h before being transferred into naïve mice. As shown in Fig 5B, the encephalitogenic CD4^+^ T cells from the GA mice induced mildly ameliorated EAE symptoms, indicating that the ameliorative effects of GA on EAE symptoms were not solely attributable to CD4^+^ T cells.

Macrophages contribute to EAE pathogenesis by producing proinflammatory mediators. Therefore, we studied the effects of GA on proinflammatory cytokines in the culture supernatant of LPS-stimulated macrophages. Consistent with the *in vivo* data, GA (580 μmol/L) suppressed the production of IL-6, IL-1β and TNF-α (Fig 5C). We also found that GA suppressed IL-23 production in response to LPS stimulation (Fig 5C). Therefore, the anti-inflammatory effects of GA on macrophages could be involved in the anti-inflammatory effects of GA on EAE.

### GA modulates the pathogenic activities of astrocytes

**Fig 6:**
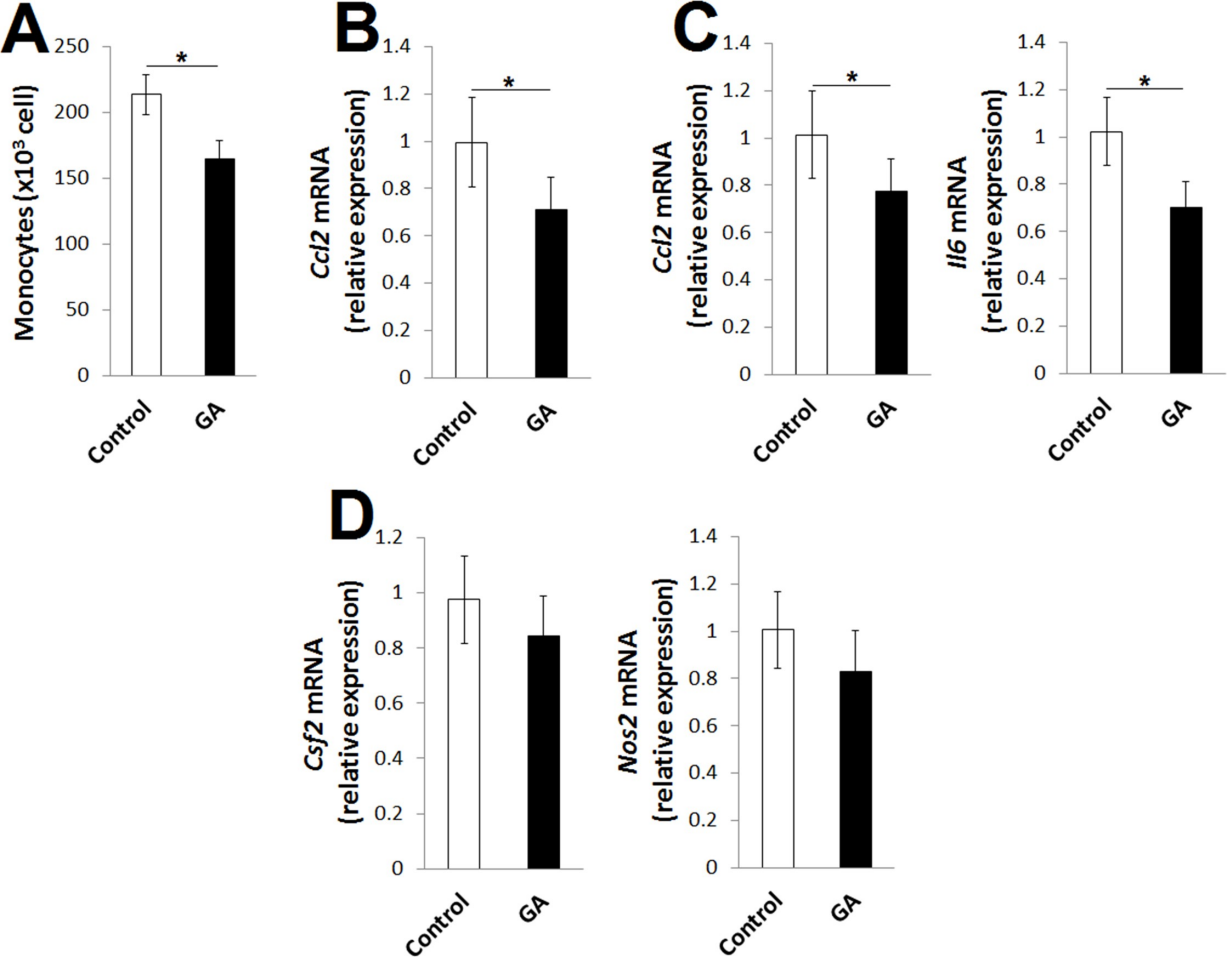
GA modulates pathogenic activities of astrocytes and microglia in EAE mice. The mice were immunized with MOG_35-55_ emulsified in CFA, and samples were collected 18 days later. The mRNA expressions of *Ccl2*, *Il6*, *Csf2 and Nos2* were assessed by quantitative real‐time PCR and normalized to *Gapdh* mRNA. (A) Absolute number of infiltrated monocytes within the CNS of control EAE (Control) and GA EAE mice. (B) Relative expression of *Ccl2* mRNA in isolated astrocytes compared to Control. (C) Relative mRNA expression of *Ccl2* and *Il6* in isolated microglia compared to Control. (D) Relative expression of *Csf2* and *Nos2* in isolated astrocytes compared to Control. Data were pooled from two independent experiments with three mice per experiment and shown as mean ± SD. *p<0.05, (one‐way ANOVA); horizontal bars denote statistical comparison.

IFN-Is in combination with Ahr ligands induce Ahr signaling in astrocytes, and this signaling attenuates CNS inflammation in EAE by suppressing colony stimulating factor 2 (*Csf2*), nitric oxide synthase 2 (*Nos2*) and monocyte chemotactic and activating factor (*Ccl2*) [21]. CCl2 exacerbates inflammation in EAE by recruiting monocytes to the site of inflammation [29]. Based on these observations, we examined whether GA affects monocyte infiltration within the CNS. The GA EAE mice showed significantly reduced numbers of infiltrating monocytes within the CNS on day 18 after MOG_35-55_ immunization (Fig 6A). This observation prompted us to quantify the mRNA expression of *Ccl2* in astrocytes from EAE mice. Interestingly, a significant reduction in the mRNA expression of *Ccl2* was found with GA treatment (Fig 6B).

Ahr signaling in astrocytes modulates the polarization and activation of microglia and monocytes [21]. Therefore, we quantified the mRNA expression of *Ccl2* and *Il6* in microglia from the CNS of EAE mice 18 days after MOG_35-55_ immunization. Interestingly, GA also repressed the mRNA expression of *Ccl2* and *Il6* in these cells (Fig 6C), suggesting that the modulatory effects of GA on the pathogenic activities of astrocytes might contribute to the ameliorative effects of GA on EAE. Notably, further investigation showed that the inhibitory effects of GA on the mRNA expression of *Csf2* and *Nos2* in astrocytes did not achieve statistical significance (Fig 6D).

## Discussion

Despite the remarkable progress in understanding the genetic control of MS [30], limited evidence is available about the roles of environmental factors [4]. In this context, Ahr exemplifies a unique link between the immune system and environment by interacting with a variety of exogenous aromatic hydrocarbons. The accumulated evidence clearly demonstrates that the activation of Ahr by exogenous ligands attenuates inflammation in a murine model of MS [19–21]. Therefore, identifying new Ahr ligands is a promising treatment strategy to control proinflammatory mediators in MS patients. Herein, we identified GA as a novel Ahr agonist of natural origin using *in silico*, *in vitro* and *in vivo* approaches.

Ahr includes two PAS domains, PAS-A and PAS-B, of which PAS-A is critically involved in Ahr/Arnt dimerization [31]. Molecular docking simulation analysis revealed the formation of five hydrogen bonds with five residues in the PAS-A domain. Importantly, three of these residues, Phe115, Leu116, and Ala119, are hydrophobic residues that play important roles in the formation of the Ahr/Arnt complex [32]. The PAS-B domain of Ahr is essential for sensing the environment and activating Ahr through interactions with ligands [31, 33, 34]. Furthermore, PAS-B plays roles in the initiation of Ahr/Arnt dimerization [33]. An analysis of the predicted interaction between PAS-B and GA revealed relatively strong interactions between PAS-B and GA. It has been demonstrated that the formation of the Ahr/Arnt heterodimer, which transforms Ahr into a transcriptionally active form, initiates the transcription of downstream genes such as *Cyp1a1* [35, 36] and the miR-212/132 cluster [8, 37]. Consistently, our data showed that GA promotes the formation of the Ahr/Arnt complex and enhances the expression of *Cyp1a1* and the miR-212/132 cluster.

A number of reports have studied the modulatory effects of GA on different aspects of the immune response [38, 39]. GA attenuates experimental colitis by suppressing IL-6, IL-1β, TNF-α, IL-17 and IFN-γ production and inhibiting p65-NF-κB and IL-6/p-STAT3 (Y705) activation [40]. GA suppresses the expression of several proinflammatory mediators, such as IL-6, IL-1β, CCL2 and CCL7, in fibroblast-like synoviocytes from rheumatoid arthritis patients [41]. However, to our knowledge, the effects of GA on the pathogenesis of autoimmune encephalomyelitis have never been investigated. In the present study, we found that the activation of Ahr by GA *in vivo* ameliorated EAE severity by suppressing the production of IL-6, IL-1β, TNF-α and IL-17 and enhancing that of TGF-β. In addition, GA reduced the frequency of CD4^+^IL-17^+^ T cells and increased that of Foxp3^+^CD4^+^ T cells. Consistently, previous studies have demonstrated that the activation of Ahr by exogenous ligands attenuates autoimmune inflammation in EAE by inhibiting IL-17-producing Th17 cells and proinflammatory cytokines and promoting the generation of TGF-β-producing Treg cells [19, 42, 43]. Several mechanisms have been proposed to elucidate the ameliorative effects of TGF-β on EAE. For instance, TGF-β suppresses the proliferation, differentiation and effector functions of encephalitogenic effector CD4^+^ T cells [44]. Furthermore, the activation of TGF-β-secreting Treg cells suppresses the infiltration of pathogenic T cells into the CNS [45]. Based on these observations, the GA-mediated increases in the TGF-β level and CD4^+^Foxp3^+^ cell frequency might have contributed to the reduced Th17 cell frequency, IL-17 production and number of infiltrating CD4^+^CD45^+^ cells in the CNS of the GA EAE mice. However, further investigation is required to identify the exact mechanism through which GA-induced TGF-β ameliorates EAE.

AChE catalyzes the breakdown of acetylcholine (ACh) and mitigates its anti-inflammatory potential. Therefore, targeting AChE augments cholinergic anti-inflammatory processes [46]. It has been demonstrated that the activation of Ahr upregulates the expression of AChE-targeting miR-132, followed by augmented cholinergic anti-inflammatory processes, in which IL-6, IL-1β, TNF-α and IL-17 levels are downregulated [11, 28]. Herein, we found that the GA EAE mice exhibited upregulation of miR-132 expression in CD4^+^ T cells and macrophages concomitant with downregulation of AChE, IL-6, IL-1β, TNF-α and IL-17 expression. Furthermore, GA inhibited AChE catalytic activity in CD4^+^ T cells and macrophages *in vitro* in a miR-132-dependent fashion, suggesting that GA could potentiate cholinergic anti-inflammatory processes. Consistent with the dual anti-inflammatory effects of GA on CD4^+^ T cells and macrophages *in vitro*, the adoptive transfer of encephalitogenic CD4^+^ T cells from the GA EAE mice induced mildly ameliorated disease symptoms. Taken together, our data indicate that GA potentiates cholinergic anti-inflammatory processes in CD4^+^ T cells and macrophages that contribute partially to the anti-inflammatory effects of GA on EAE.

Interestingly, we found that GA suppressed the expression of *Ccl2* in astrocytes and microglia from EAE mice. Recent supporting observations have shown that Ahr controls the proinflammatory functions of astrocytes during EAE by limiting the recruitment of NF-κB to the promoters of responding factors such as *Ccl2*, *Csf2* and *Nos2* [21]. CCl2 is implicated in the recruitment of monocytes [47] and T cells [48] to the site of inflammation. Therefore, the reduced expression of *Ccl2* in these CNS-resident cells might have contributed to the reduced number of infiltrating monocytes and CD4^+^CD45^+^ T cells. Moreover, the reduced number of infiltrating monocytes could be a part of the GA-potentiated cholinergic anti-inflammatory processes. This suggestion is supported by recent findings showing that the ligation of nicotinic acetylcholine receptor inhibits the infiltration of monocytes and neutrophils into the CNS and suppresses the mRNA expression of *Ccl2* and chemokine C-X-C motif ligand 2 (*CXCL2*) in the brain [49]. It has been demonstrated that Ahr signaling in astrocytes regulates their neurotoxic activities during EAE, the activation of microglia and monocytes and the recruitment of monocytes to the CNS [21]. These observations may partially explain the reduced expression of *Il6* in microglia. Notably, IL-6 and IL-23a are associated with differentiation and effector functions in Th17 cells [50]. Overall, the cholinergic anti-inflammatory processes and modulation of the pathogenic activity of astrocytes might have contributed to the anti-inflammatory effects of GA on EAE.

In summary, our findings identify GA as a novel Ahr ligand of natural origin and clearly indicate that GA has anti-inflammatory properties in EAE. The mechanisms underlying the ameliorative effects of GA on EAE involve promoting Treg cell generation, potentiating miR-132-mediated cholinergic anti-inflammatory processes and modulating the pathogenic activities of astrocytes. These findings suggest that GA is a promising candidate to control inflammation in autoimmune diseases such as MS.

## Supporting information

S1 Fig *In silico* molecular modeling of mouse Ahr PAS-B domain. A) Superimposition of predicted 3D structure of mouse Ahr PAS-B domain with the human C-terminal PAS domain of HIF2a (PDB ID: 1p97). (B) Ramachandran plot of predicted mouse Ahr PAS-B domain.
(PDF)

S2 Fig Efficiency of cell stimulation milieus. The CD4^+^CD62L^+^ T cells were isolated from the spleen and cultured under Th17-, Treg or Tr1-polarizing conditions, and peritoneal macrophages were cultured in presence of LPS. The mRNA expression of *Rorc*, *Il6*, *FoxP3* and *Il10* were assessed by quantitative real‐time PCR and normalized to *Gapdh* mRNA. (A) Relative expression of *Rorc* in CD4^+^CD62L^+^ T cells cultured under Th17-conditions for 48 hr compared to Th0. (B) Relative expression of *Il6* mRNA in macrophages stimulated with LPS for 4 hr. (C) Relative expression of *FoxP3* mRNA in CD4^+^CD62L^+^ T cells cultured under Treg-polarizing conditions for 48 hr. (D) Relative expression of *Il10* mRNA in CD4^+^CD62L^+^ T cells cultured under Tr1-polarizing conditions for 56 hr. Data were pooled from independent experiments and shown as mean ± SD.
(PDF)

S3 Fig High levels of GA show toxic effects. (A and B) The CD4^+^CD62L^+^ T cells were isolated from the spleen and cultured under Th17-polarizing conditions, and peritoneal macrophages were cultured in presence of LPS. Cell viability of (A) differentiating Th17 and (B) peritoneal macrophages 48 hr after stimulation in presence of GA (20–120 μmol/L). (C and D) The EAE was induced by immunizing mice with MOG_35-55_ emulsified in CFA. The mice were injected intraperitoneally with vehicle (corn oil) or GA (1–4 mg/day) for 14 days starting one day before MOG_35-55_ immunization. Weight of (C) spleen and (D) liver were measured 24 h after last dose, *n* = 6. Data were pooled from independent experiments and shown as mean ± SD. *p < 0.05.
(PDF)

S4 Fig GA suppresses AChE activity in CD4^+^ T cells and macrophages. AChE catalytic activity in culture supernatant of (A) CD4^+^ T cells isolated from naive mice and stimulated with PHA and (B) peritoneal macrophages were stimulated with LPS. The PHA-stimulated CD4^+^ T cells and LPS-stimulated macrophages were electroporated with antisense (as)-miR-132, and cells treated with PHA, PHA+GA, LPS and LPS+GA were electroporated with scramble hairpin inhibitor. Data were pooled from independent experiments and shown as mean ± SD. *p < 0.05, PHA+GA versus PHA, and LPS+GA versus LPS; ǂp < 0.05, PHA+GA+as-miR-132 versus PHA, and LPS+GA+as-miR-132 versus LPS; #p < 0.05, PHA+GA versus PHA+GA+as-miR-132, and LPS+GA versus LPS+GA+as-miR-132.
(PDF)
