# Correction: Activation of aryl hydrocarbon receptor signaling by a novel agonist ameliorates autoimmune encephalomyelitis

**DOI:** 10.1371/journal.pone.0223429

**Published:** 2019-10-10

**Authors:** The PLOS ONE Staff

The authors’ given names and surnames are switched. The correct names are: Abdullah Alzahrani, Maged Mohammed, Hairul-Islam M. Ibrahim, Osama I. Alwassil, Maha Habash, Manal Alfuwaires, Hamza Hanieh. The correct citation is: Alzahrani A, Mohammed M, Ibrahim HM, Alwassil OI, Habash M, Alfuwaires M, et al. (2019) Activation of aryl hydrocarbon receptor signaling by a novel agonist ameliorates autoimmune encephalomyelitis. PLoS ONE 14(4): e0215981.https://doi.org/10.1371/journal.pone.0215981. The publisher apologizes for the errors.
