# Supplementary material for: Activation of aryl hydrocarbon receptor signaling by a novel agonist ameliorates autoimmune encephalomyelitis
Source: PLoS One. 2019 Apr 26;14(4):e0215981. doi: 10.1371/journal.pone.0215981 (PMC6485712; doi:10.1371/journal.pone.0215981)
Supplement: S1 Fig — A) Superimposition of predicted 3D structure of mouse Ahr PAS-B domain with the human C-terminal PAS domain of HIF2a (PDB ID: 1p97). (B) Ramachandran plot of predicted mouse Ahr PAS-B domain. (PDF) [file pone.0215981.s001.pdf]

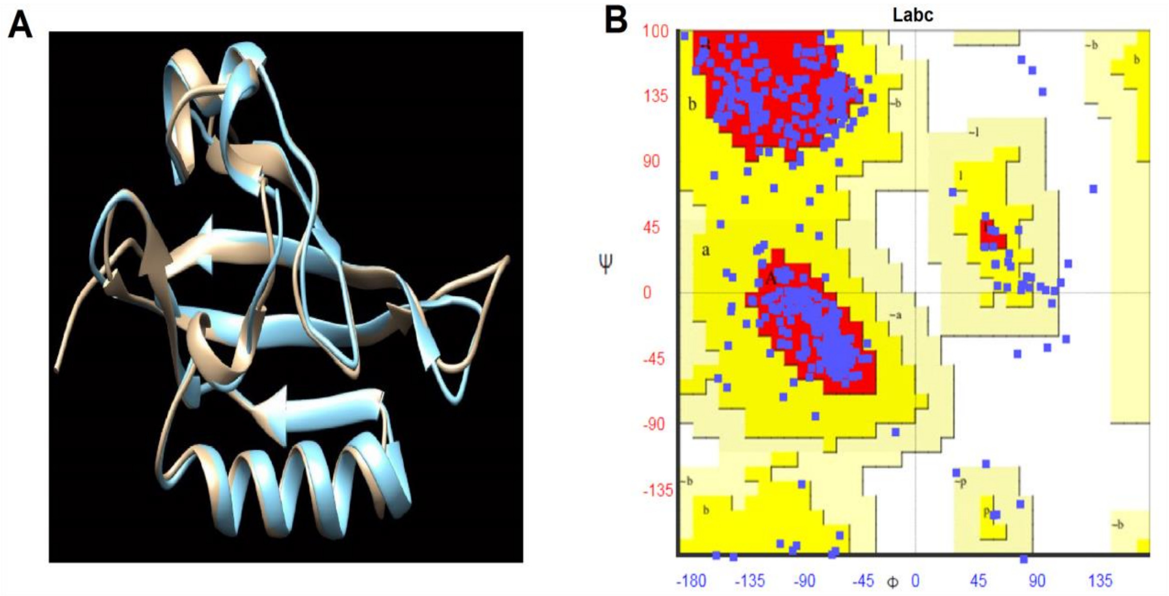


**S1 Fig. *In silico* molecular modeling of mouse Ahr PAS-B domain.** A) Superimposition of predicted 3D structure of mouse Ahr PAS-B domain with the human C-terminal PAS domain of HIF2a (PDB ID: 1p97). (B) Ramachandran plot of predicted mouse Ahr PAS-B domain.
