# Supplementary material for: Activation of aryl hydrocarbon receptor signaling by a novel agonist ameliorates autoimmune encephalomyelitis
Source: PLoS One. 2019 Apr 26;14(4):e0215981. doi: 10.1371/journal.pone.0215981 (PMC6485712; doi:10.1371/journal.pone.0215981)
Supplement: S2 Fig — The CD4+CD62L+ T cells were isolated from the spleen and cultured under Th17-, Treg or Tr1-polarizing conditions, and peritoneal macrophages were cultured in presence of LPS. The mRNA expression of Rorc, Il6, FoxP3 and Il10 were assessed by quantitative real‐time PCR and normalized to Gapdh mRNA. (A) Relative expression of Rorc in CD4+CD62L+ T cells cultured under Th17-conditions for 48 hr compared to Th0. (B) Relative expression of Il6 mRNA in macrophages stimulated with LPS for 4 hr. (C) Relative expression of FoxP3 mRNA in CD4+CD62L+ T cells cultured under Treg-polarizing conditions for 48 hr. (D) Relative expression of Il10 mRNA in CD4+CD62L+ T cells cultured under Tr1-polarizing conditions for 56 hr. Data were pooled from independent experiments and shown as mean ± SD. (PDF) [file pone.0215981.s002.pdf]

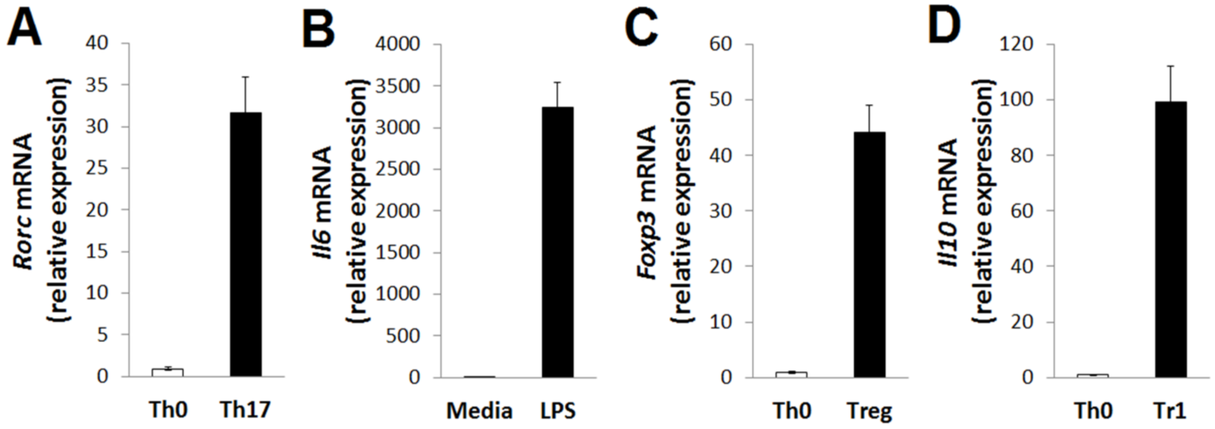

S2 Fig. Efficiency of cell stimulation milieus. The $CD4^+CD62L^+$ T cells were isolated from the spleen and cultured under Th17-, Treg or Tr1-polarizing conditions, and peritoneal macrophages were cultured in presence of LPS. The mRNA expression of *Rorc*, *Il6*, *FoxP3* and *Il10* were assessed by quantitative real-time PCR and normalized to *Gapdh* mRNA. (A) Relative expression of *Rorc* in $CD4^+CD62L^+$ T cells cultured under Th17-conditions for 48 hr compared to Th0. (B) Relative expression of *Il6* mRNA in macrophages stimulated with LPS for 4 hr. (C) Relative expression of *FoxP3* mRNA in $CD4^+CD62L^+$ T cells cultured under Treg-polarizing conditions for 48 hr. (D) Relative expression of *Il10* mRNA in $CD4^+CD62L^+$ T cells cultured under Tr1-polarizing conditions for 56 hr. Data were pooled from independent experiments and shown as mean ± SD.
