# Supplementary material for: Activation of aryl hydrocarbon receptor signaling by a novel agonist ameliorates autoimmune encephalomyelitis
Source: PLoS One. 2019 Apr 26;14(4):e0215981. doi: 10.1371/journal.pone.0215981 (PMC6485712; doi:10.1371/journal.pone.0215981)
Supplement: S3 Fig — (A and B) The CD4+CD62L+ T cells were isolated from the spleen and cultured under Th17-polarizing conditions, and peritoneal macrophages were cultured in presence of LPS. Cell viability of (A) differentiating Th17 and (B) peritoneal macrophages 48 hr after stimulation in presence of GA (20–120 μmol/L). (C and D) The EAE was induced by immunizing mice with MOG35-55 emulsified in CFA. The mice were injected intraperitoneally with vehicle (corn oil) or GA (1–4 mg/day) for 14 days starting one day before MOG35-55 immunization. Weight of (C) spleen and (D) liver were measured 24 h after last dose, n = 6. Data were pooled from independent experiments and shown as mean ± SD. *p < 0.05. (PDF) [file pone.0215981.s003.pdf]

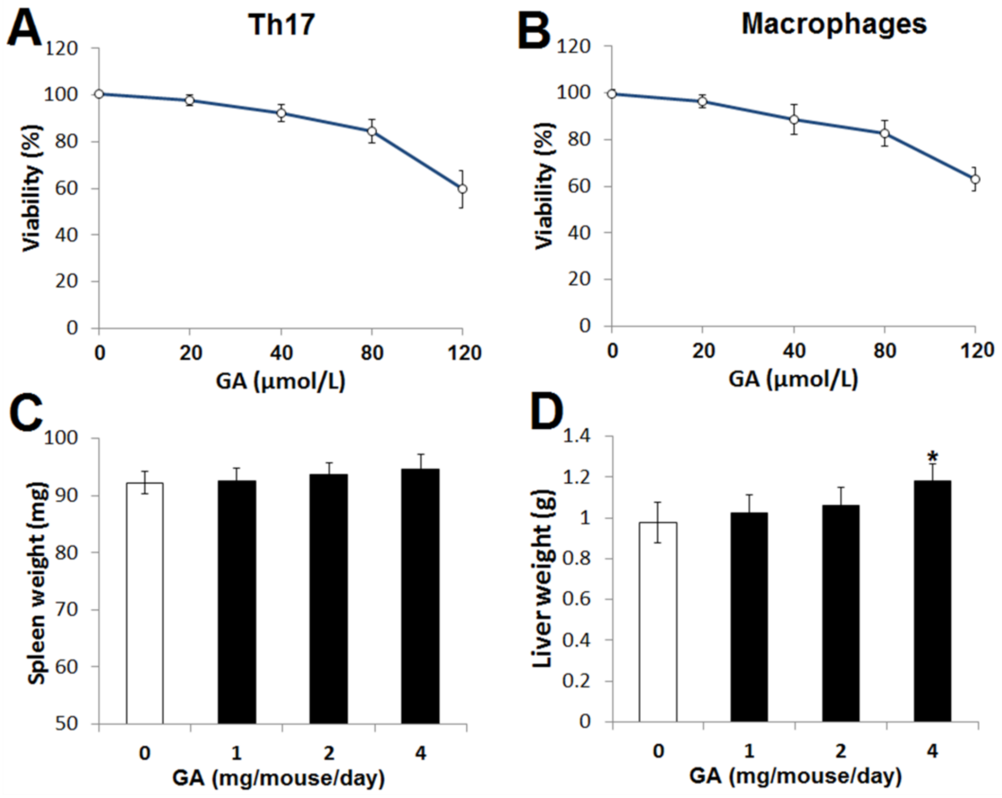

S3 Fig. High levels of GA show toxic effects. (A and B) The $CD4^{+}CD62L^{+}$ T cells were isolated from the spleen and cultured under Th17-polarizing conditions, and peritoneal macrophages were cultured in presence of LPS. Cell viability of (A) differentiating Th17 and (B) peritoneal macrophages 48 hr after stimulation in presence of GA (20-120 μmol/L). (C and D) The EAE was induced by immunizing mice with $MOG_{35\text{-}55}$ emulsified in CFA. The mice were injected intraperitoneally with vehicle (corn oil) or GA (1-4 mg/day) for 14 days starting one day before $MOG_{35\text{-}55}$ immunization. Weight of (C) spleen and (D) liver were measured 24 h after last dose, n=6. Data were pooled from independent experiments and shown as mean ± SD. *$p < 0.05$.
