# Supplementary material for: Activation of aryl hydrocarbon receptor signaling by a novel agonist ameliorates autoimmune encephalomyelitis
Source: PLoS One. 2019 Apr 26;14(4):e0215981. doi: 10.1371/journal.pone.0215981 (PMC6485712; doi:10.1371/journal.pone.0215981)
Supplement: S4 Fig — AChE catalytic activity in culture supernatant of (A) CD4+ T cells isolated from naive mice and stimulated with PHA and (B) peritoneal macrophages were stimulated with LPS. The PHA-stimulated CD4+ T cells and LPS-stimulated macrophages were electroporated with antisense (as)-miR-132, and cells treated with PHA, PHA+GA, LPS and LPS+GA were electroporated with scramble hairpin inhibitor. Data were pooled from independent experiments and shown as mean ± SD. *p < 0.05, PHA+GA versus PHA, and LPS+GA versus LPS; ǂp < 0.05, PHA+GA+as-miR-132 versus PHA, and LPS+GA+as-miR-132 versus LPS; #p < 0.05, PHA+GA versus PHA+GA+as-miR-132, and LPS+GA versus LPS+GA+as-miR-132. (PDF) [file pone.0215981.s004.pdf]

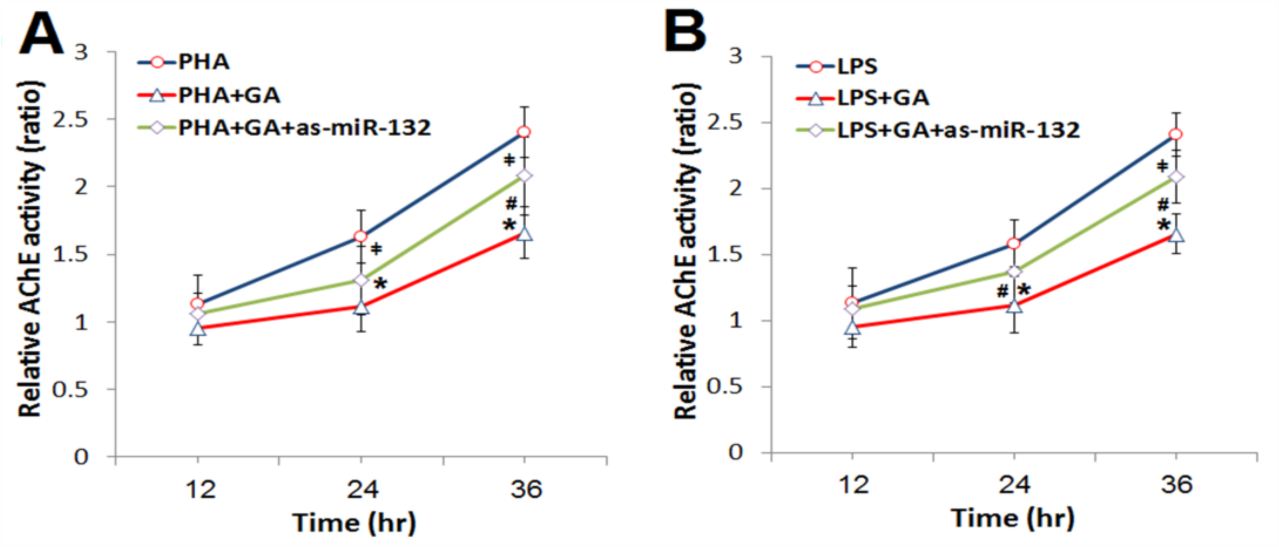

S4 Fig. GA suppresses AChE activity in CD4$^+$ T cells and macrophages. AChE catalytic activity in culture supernatant of (A) CD4$^+$ T cells isolated from naive mice and stimulated with PHA and (B) peritoneal macrophages were stimulated with LPS. The PHA-stimulated CD4$^+$ T cells and LPS-stimulated macrophages were electroporated with antisense (as)-miR-132, and cells treated with PHA, PHA+GA, LPS and LPS+GA were electroporated with scramble hairpin inhibitor. Data were pooled from independent experiments and shown as mean ± SD. *$p < 0.05$, PHA+GA versus PHA, and LPS+GA versus LPS; ǂp < 0.05, PHA+GA+as-miR-132 versus PHA, and LPS+GA+as-miR-132 versus LPS; #p < 0.05, PHA+GA versus PHA+GA+as-miR-132, and LPS+GA versus LPS+GA+as-miR-132.
